# Double Amputation of Lower Extremities for Gangrene Following Frost Bite

**Published:** 1892-04

**Authors:** H. B. Hill

**Affiliations:** Austin, Texas


For Daniel's Texas Medical Journal.

### DOUBLE AMPUTATION OF LOWER EXTREMITY FOR GANGRENE FOLLOWING FROST BITE.

H. B. HILL, M. D., AUSTIN, TEXAS.

[Read at Austin District Medical Society.]

ALTHOUGH the following case occurred several years ago, I thought the rarity of cause of the condition, in this climate, would justify me in reporting it. It rarely ever occurs here, except from exposure while "dead drunk."

The subject of this sketch, Lee Curry, was a small negro boy, about ten years old, and of the genus known in common parlance as "a natural born thief." In the early part of January, 1888, he left his mother's house, and went to the home of one Jim Christian, a negro, whose reputation for honesty was down to about zero. While there he engaged in a number of petty thieveries, the elder negro playing the part of receiver for the stolen property. When their operations were discovered, the negro Jim, to remove suspicion from himself, in an apparent fit of indignation, drove the boy away from his home, about evening of January 19, 1888. The boy, not going very far, returned to the place after dark, and crept into an open rail pen, in which there was a small quantity of corn and fodder, with which he partly covered his body, but leaving his feet and the lower portion of his legs exposed, without shoes or stockings. About 9 o'clock that night the coldest spell of weather I have ever experienced in Texas, made its appearance, and to which this boy was exposed from Saturday night until late in the afternoon the following Monday. At that time he was found by the negro Jim, in an insensible condition, with an ear of corn frozen fast to each foot. He carried him into the house, and immersed his feet into very hot water, and allowed them to remain until they were completely thawed. Of course, this was the worst thing that he could have done, and made the loss of his feet a certainty. He remained at this house for one week, and then was put in an open wagon and hauled the distance of ten miles, where he remained three days longer before I was summoned, eleven days having elapsed since his exposure without any medical attention whatever. I found that complete gangrene of both feet and a portion of each leg had occurred, a few strokes of the scissors sufficing to remove the entire mass at the ankle joints, leaving the bones of both legs denuded of periosteum for several inches. As it was about sunset when I first saw him, I deferred operating until the next morning, merely cleansing the parts thoroughly and applying an anti-septic dressing. The next morning, with the assistance of my friend Dr. H. Upshaw, of Gay Hill, Texas, and Mr. Bohmes, an intelligent non-professional, I amputated both legs.

The only question to be decided was the site of the operation, as the line of demarcation was well defined. We decided to amputate both limbs at the same point, a short distance below the tuberosity of the tibia, as the poverty of the patient precluded the possibility of his getting complicated artificial limbs. As the patient was quite weak, I administered a full dose of digitalis in a a stiff whiskey toddy one-half hour before commencing the operation. The anæsthetic used was chloroform, and the antiseptics, bichloride of mercury aud carbolic acid. The posterior flaps were made by transfixion, and the anterior by cutting from without inwards. As we were not supplied with an Esmarch bandage, I improvised a very efficient substitute, by first applying very tightly an ordinary roller bandage until it reached a short distance above the knee, and then taking several turns around the limb with several feet of rubber tubing, and tying the ends into a common bow-knot. This simple device was so successful that the patient did not lose more than one ounce of blood during the entire double operation. We used strict antiseptic precautions, and at the close of the operation, the pulse was rather better than at the beginning; probably due to the effects of the whiskey and digitalis. The stumps were enveloped fully with sterilized cotton-batting, and other antiseptic dressings, which were not disturbed for ten days, when there was found to be almost complete union of the flaps. The subsequent history of the case may be briefly summarized. There was not a bad symptom during the entire progress. The temperature never rose above 100°, and he soon greedily devoured what food was allowed him.

The site of the amputation proved to have been well chosen, as it was found that by turning the heel of a shoe forward, it fitted the stump very well, and he walked on his knees very successfully.

I suggested to the boy's mother, that perhaps the loss of his limbs would be fortunate for him, and be the means of preventing him from leading a life of crime, but such was not the case. Two years after the operation, he, with the assistance of a younger brother, stole four horses, two saddles, bridles, halters, etc., and was arrested in the town of Brenham, while attempting to sell them, and is now an inmate of the State reformatory, at Gatesville.

